# Six Months Guided Exercise Therapy Improves Motor Abilities and White Matter Connectivity in Children with Cerebral Palsy

**DOI:** 10.21315/mjms2020.27.5.9

**Published:** 2020-10-27

**Authors:** Md Safwan Samsir, Rahimah Zakaria, Salmi Abdul Razak, Mohamed Saat Ismail, Mohd Zulkifli Abdul Rahim, Chia-Shu Lin, Nik Mohammad Faez Nik Osman, Mohammad Afiq Asri, Nor Haslina Mohd, Asma Hayati Ahmad

**Affiliations:** 1 Department of Physiology, School of Medical Sciences, Universiti Sains Malaysia, Kubang Kerian, Kelantan, Malaysia; 2 Faculty of Psychology and Education, Universiti Malaysia Sabah, Malaysia; 3 Department of Paediatrics, School of Medical Sciences, Universiti Sains Malaysia, Kubang Kerian, Kelantan, Malaysia; 4 School of Health Sciences, Universiti Sains Malaysia, Kubang Kerian, Kelantan, Malaysia; 5 Department of Dentistry, School of Dentistry, National Yang-Ming University, Taipei, Taiwan; 6 Hospital Universiti Sains Malaysia, Kubang Kerian, Kelantan, Malaysia

**Keywords:** cerebral palsy, exercise therapy, diffusion MRI

## Abstract

**Background:**

Diffusion magnetic resonance imaging (dMRI) provides the state of putative connectivity from lesioned areas to other brain areas and is potentially beneficial to monitor intervention outcomes. This study assessed the effect of a 6 months guided exercise therapy on motor abilities and white matter diffusivity in the brains of cerebral palsy (CP) children.

**Methods:**

This is a single arm pre-and post-test research design involving 10 spastic CP children, aged 8–18 years and whose Gross Motor Function Classification System Expanded and Revised (GMFCS-E & R) at least Level 21 with the ability to ambulate independently. They were recruited from Paediatric Neurology Clinic, Hospital Universiti Sains Malaysia (HUSM) from December 2015–December 2016. All participants underwent 6 months of therapist-guided exercise session comprising progressive strength training at a frequency of twice a week, 1 h duration per session. The effect of exercise on motor abilities was assessed using the Gross Motor Function Measures (GMFM)-88. Six out of the 10 children consented for dMRI. Probabilistic tractography of the corticospinal tract (CST) was performed to determine the connectivity index of the tracts pre-and post-intervention.

**Results:**

All the participants displayed statistically significant increment in GMFM-88 scores pre-to post-exercise intervention. This improvement was concurrent with increased connectivity index in the CST of upper limbs and lower limbs in the brain of these children.

**Conclusion:**

Our findings demonstrated that 6 months guided exercise therapy improves motor abilities of CP children concurrent with strengthening the connectivities of the motor pathways in the brain.

## Introduction

Cerebral palsy (CP) patients experience a variety of muscle dysfunctions such as muscle spasticity, loss of selective muscle control and muscle weakness (1). Exercise has been shown to reduce muscle spasticity and increase muscle strength. Even though motor skills of most CP patients eventually improve as they grow, adults with CP are at risk of decreased mobility and health complications if left untreated. An exercise therapy may consist of several types of training such as strength training, fitness training, static weight bearing and passive stretching (2). Though it has been proven to have its effect on muscular strength, volume and significantly increase motor ability of patients with CP, the type and duration of exercise therapy programme that should be prescribed to these patients remain unclear (3).

There have been several diffusion magnetic resonance imaging (dMRI) studies in CP patients demonstrating damage to white matter tracts throughout the brain (4), differences in the white matter tract damage comparable to the types of CP (5), as well as diffuse connectivity deficits associated with severity of functional impairment (6, 7) in cross sectional studies. Furthermore, CP is a heterogeneous disorder with multiple causes and clinical manifestations, meaning that the specific structural changes that may underlie improved function are likely to be unique to each patient. Brain structural connectivity has been shown to increase concurrent with functional improvement in CP children receiving autologous cord blood transfusions (with order randomised with a placebo administered over 2 years) in conjunction with standard physical and occupational therapies (8). Constraint-induced movement therapy has also been shown to potentially induce changes in corticospinal tract (CST) in CP children under the age of 2 (9).

Recently our group reported that dMRI with probabilistic tractography provides the state of connectivity from lesioned areas to other parts of the brain and is potentially beneficial to be used as a means to monitor intervention outcomes and as an adjunct to the clinical management of CP (10). Thus, the present study aimed to examine the effect of a 6-month exercise therapy on motor abilities of CP children using the Gross Motor Function Measures (GMFM) and on white matter diffusivity using dMRI with probabilistic tractography.

## Methods

**Figure 1:**
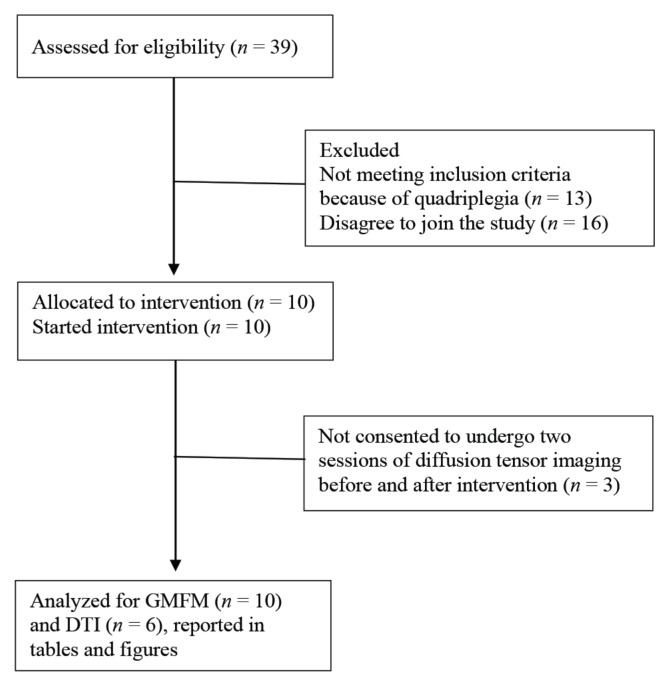
Modified CONSORT flowchart for a single-arm non-randomised, own-control study of guided exercise therapy in children with CP

This study was conducted from December 2015–December 2016. A within-subjects, single arm pre- and post-test research design was employed. The effect of exercise on motor abilities was assessed using the GMFM-88 administered pre- and post-exercise therapy, while the effects on white matter diffusivity were assessed using dMRI and probabilistic tractography. The modified Consolidated Standards of Reporting Trials (CONSORT) flowchart is provided in Figure 1.

### Participants

Ten spastic CP children with age ranging between 8 and 18 years old (mean age ± SD = 13.4 ± 3.307, six males) were recruited (Table 1). Six of the children had bilateral and four had unilateral CP. All potential participants had been evaluated and diagnosed as CP by a pediatric neurologist. All participants were on follow-up in Paediatric Neurology Clinic, Hospital Universiti Sains Malaysia (HUSM). A total of 39 CP children data were retrieved from the Record Department of HUSM within the given age range. Thirteen children with quadriplegia were excluded from this study. Out of the remaining potential participants, only 10 (*n* = 10) children fulfilled the inclusion/exclusion criteria and their parents or guardian agreed to participate in the exercise therapy programme. Out of the 10 participants, 6 parents or guardian consented to undergo two sessions of diffusion tensor imaging (DTI) before and after intervention. Exercise sessions were performed in the participants’ houses while scanning was done in the Department of Radiology HUSM.

Inclusion criteria included age between 8 and 18 years old, medical follow-up of at least 6 months, written informed consent from parents/guardian, and presence of hand function, at least able to grasp object. Meanwhile, in terms of mobility, children should at least be Level 2 (children could walk and run independently, despite reduced balance, speed and coordination) in GMFM, and able to ambulate independently without assistive device. Children were excluded if they have history of orthopaedic surgery or neurosurgery, presence of any diagnosed genetic syndrome or having active seizures 6 months prior to exercise intervention, had received botulinum toxin injections within the past 6 months and had marked intellectual disability and inability to follow instructions. Physically, presence of muscle or tendon contracture, presence of dystonia or athetoid were also criteria for exclusion. Additional exclusion criteria for magnetic resonance imaging (MRI) scanning was having metal implants in the body.

### Study Procedure

Participants underwent individually-guided exercise therapy by a well-trained exercise instructor who came to each child’s home or school to conduct the exercise therapy sessions. The structured programme was implemented and conducted as after-school exercise sessions. The children underwent 6 months of exercise therapy at a frequency of twice a week, and duration of 1 h each session.

A session consisted of 5 min warm-up, 45 min workout session and another 5 min of cool-down session in a circuit training way. Each work station was set up for intensive repetitive practice of an exercise practising. Children were moved between stations, practicing functional based exercise such as step-ups, sit-to-stand and some other exercise movement. Resistance or load was increased progressively as strength increased.

Participants had GMFM scoring as baseline prior to exercise therapy and after completion of 6 months exercise therapy. Six participants who provided informed consent also, additionally, underwent MRI scans of the brain prior and post exercise therapy.

### Exercise Therapy

The exercise regime was developed according to the needs of the CP children and administered in accordance with the child’s strength and ability. Nevertheless, the training programme was standardised in terms of protocol and duration (11). This regime included exercise regime that mainly focused on muscle strength. Exercises were administered using the periodisation as well as the reversibility principles (Table 2).

Strength training exercises were prescribed individually and by referring to the periodisation table (Table 2). During the early phase of training (anatomical adaptation), the volume of exercise was high while the intensity was low. For example, in the initial phase of step-up exercise, the children may step-up on the step without any weight but the number of repetition was high. Later in the exercise phase, they were gradually made to wear weights (wearing weight vest or ankle strap weight) while doing the exercise. They were started with 30% of their maximum capacity as recommended by previous studies (1, 12). In the maximum strength phase, the volume of exercise was reduced by reducing the number of repetition per set of exercise. The intensity of exercise was increased to 50%–65% of their capacity and gradually increased with time.

The reversibility principle of training was applied during the exercise therapy whereby the amount of load or intensity of training were decreased according to the children’s capacity. For example, if the child skipped the exercise because of sickness, their capabilities would decrease. Thus, the intensity of training also needed to be decreased (1).

## Functional Outcome Measures

The GMFM consists of 88 items that are grouped into five dimensions of gross motor function; i) lying and rolling (17 items); ii) sitting (20 items); iii) crawling and kneeling (14 items); iv) standing (13 items); and v) walking, running and jumping (24 items). Each item from the five dimensions is scored based on a 4-point Likert scale. A percentage is calculated for each dimension as described by Ketelaar et al. (13).

To minimise research bias, GMFM was administered by researchers who were not directly related to this study. Researchers were among exercise’s instructors that recruited and provided with a scoring manual book to ensure the scores were given objectively and the examination session was video recorded. GMFM test was administered before commencement of exercise therapy and 6 months after therapy had been completed. The tests were done at the participants’ homes or schools.

### DTI

MRI scanning of the brain for DTI was performed pre- and post-intervention on six participants who provided informed consent. Before proceeding with the DTI scanning, sedation was given to minimise vigorous head motion during the scanning process. Sedation was administered by a medical officer and they were not allowed to consume food 5 h–6 h prior to sedation. Initially, oral chloral hydrate (50 mg/kg/dose–75 mg/kg/dose) was given. If the children did not show positive reaction, intravenous midazolam (0.1 mg/kg/dose) or intravenous ketamine (1 mg/kg/dose) was used. Intravenous atropine (0.01 mg/kg/dose) was given to decrease secretion for those who were given ketamine. To ensure children safety and minimising other complication during the sedation, they were warded and continuously monitored using SpO2 and cardiac monitoring until permitted to be discharged.

dMRI scan was obtained using Philips Achieva 3.0T system with 32-channel SENSE head coil. Imaging parameters were as follows: matrix = 128 × 128, field of view = 221 mm × 221 mm, repetition time/echo time = 10,726/76 ms, SENSE factor = 2; EPI factor = 67 and *b* = 1000 mm^2^ s^−1^, NEX = 1 and thickness = 2.3 mm for each of the 32 non-colinear diffusion-sensitising gradients. Raw imaging data in HDR form was converted to 4D NIfTi form using the MRIcron dcm2nii dicom to niftii converter software to render it ready for further processing.

### Drawing of Regions of Interest Masks

Drawing of regions of interest (ROI) masks were hand-drawn, starting in one view and double-checked in multiplanar views using FSLview in FMRIB Software Library (FSL, www.fmrib.ox.ac.uk/fsl ). Seed masks were the motor area for upper extremities in the pre-central knob and the mediodorsal part of the precentral gyrus for the motor area for lower extremities. Mask for the target region was drawn in the lower pons. Seed and target masks were chosen to trace the CST pathway from the upper limb and lower limb area in the motor cortex descending to the pons.

### Probabilistic Tractography

Diffusion data were pre-processed using FMRIB’s Diffusion Toolbox (FDT, http://fsl.fmrib.ox.ac.uk/fsl/fdt ) (14, 15) in FSL. Head motion effect and image distortion due to eddy currents were corrected using eddy_correct. Bayesian Estimation of Diffusion Parameters Obtained using Sampling Techniques (BEDPOSTX) in FDT was then run on imaging data that have been eddy-corrected. BEDPOSTX runs Markov Chain Monte Carlo sampling to build up distributions on diffusion parameters at each voxel.

Probabilistic tractography was performed on diffusion-weighted bedpostx datasets using probtrackx in FDT according to previously described methods (14, 16). Using Bayesian principle, a probability diffusion function was estimated for every voxel to determine the principal fiber direction. Five thousand streamline samples were generated from each seed voxel to build up a connectivity distribution in diffusion space. The number of these samples passing through each brain voxel is proportional to the connection probability of the seed voxel.

For each subject, probabilistic connectivity distribution from all voxels in each seed region to the target area was quantified. This gave the mean number of samples per voxel in the seed area with positive connection probability to the target area. The mean per voxel multiplied by the volume (number of voxels) with positive connection probability gives the connectivity index.

### Statistical Analysis

A priori sample size calculation indicated that six participants were needed to detect a clinically significant difference of training effect on white matter integrity. Power was set at 90%, significance level at 5% and the standard deviation of the difference was set at 0.03 (11). To allow for loss to follow-up and consent for DTI, 10 children were included in this study. Results were analysed using IBM SPSS version 22. Comparison of overall GMFM scores between pre- and post-intervention was calculated using one-tailed paired *t*-test. GMFM scores for each dimension and connectivity index output from tractography were analysed using repeated-measures ANOVA. For GMFM, within-subject factors TYPE (five levels: Lying, Sitting, Crawling, Standing and Walking) and TIME (two levels: pre- and post-intervention) were used; while for connectivity index, within-subject factors were AREA (four levels: left lower limb, left upper limb, right lower limb and left upper limb) and TIME (two levels: pre- and post-intervention). Greenhouse-Geisser corrections were applied when assumption of sphericity was not met in Mauchly’s test. Post-hoc tests were performed using one-tailed paired *t*-tests with Bonferroni corrected for multiple comparisons.

## Results

All the CP children displayed improvement in their abilities to perform the GMFM after undergoing exercise therapy for 6 months. During the first 2 weeks of therapy, some of the younger children were not really committed with their exercise regime. But as time went on, with motivation and support from the instructor, friends and family, they started to focus and had positive progress of their exercise therapy.

### GMFM

**Figure 2:**
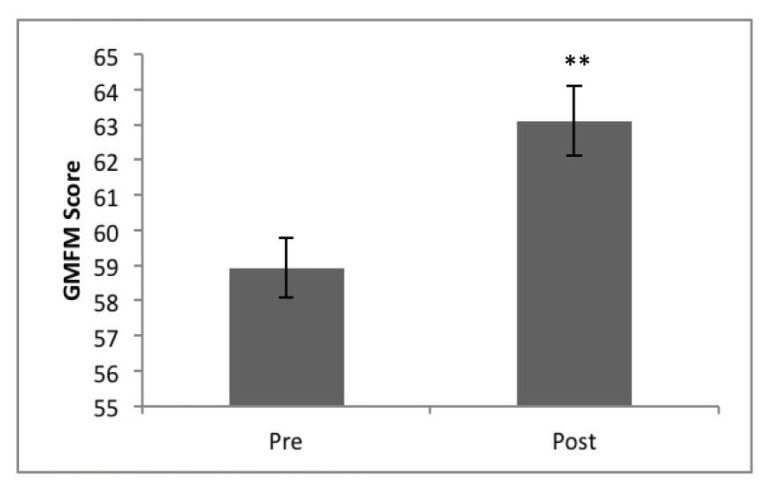
Overall GMFM scores measured pre- and post-exercise therapy intervention Notes: Data are mean ± SEM. *P < 0.05, **P < 0.01, ***P < 0.005

The results of one tailed paired *t*-test for GMFM scores are shown in Table 3. Overall, the GMFM score showed significantly higher postintervention scores compared to preintervention, *t*(9) = −11.7, *P* < 0.001, *r* = 0.97 (Figure 2). Repeated measures ANOVA for the different dimensions of GMFM showed significant main effect of TYPE, *F*(4, 36) = 126.6, *P* < 0.001, indicating significantly different scores among the types of dimensions. There was also significant main effect of TIME, *F*(1, 9) = 129.8, *P* < 0.001.

**Figure 3:**
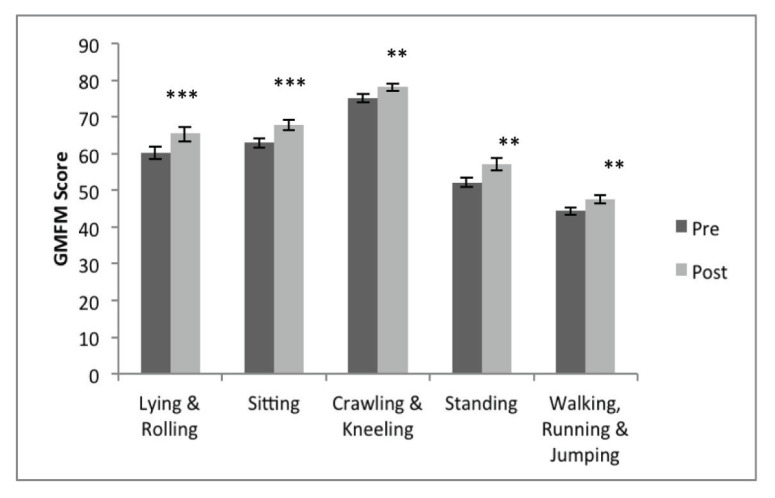
GMFM dimensions measured pre- and post-exercise therapy intervention Notes: Data are mean ± SEM. *P < 0.05, **P < 0.01, ***P < 0.005

Post-hoc paired *t*-test, one tailed, adjusted for multiple comparisons at significance level 0.01 showed post-intervention GMFM were higher for all dimensions (Figure 3); Lying and Rolling, *t*(9) = −6.5, *P* < 0.001, *r* = 0.91; Sitting, *t*(9) = −6.3, *P* < 0.001, *r* = 0.9; Crawling, *t*(9) = −5.9, *P* < 0.001, *r* = 0.89; Standing, *t*(9) = −3.8, *P* = 0.002, *r* = 0.78; and Walking, Running and Jumping, *t*(9) = −4.1, *P* = 0.001, *r* = 0.81. Lying and Rolling dimension had the highest improvement rate at 5.1% followed by Standing (4.9%), Sitting (4.8%), Crawling and Kneeling (3.1%) and lastly, Walking, Running and Jumping (3.1%).

### Tractography

Repeated measures ANOVA showed significant main effects of AREA, *F*(3, 15) = 7.6, *P* = 0.003 indicating that the connectivity index was different among the CST of the upper limb and lower limb bilaterally. There was also significant main effect of TIME, *F*(1, 5) = 16.9, *P* = 0.009 indicating differences between pre-and post-intervention connectivity indices. Significant interaction was also found between AREA and TIME, *F*(3, 15) = 5.6, *P* = 0.009.

**Figure 4:**
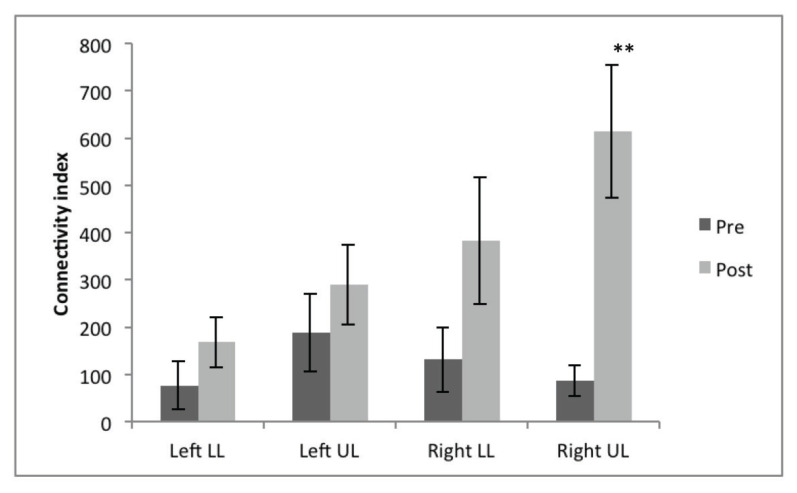
Connectivity index pre- and post-intervention in the left and right upper limbs and lower limbs Notes: *P < 0.05, **P < 0.01, ***P < 0.005

**Figure 5:**
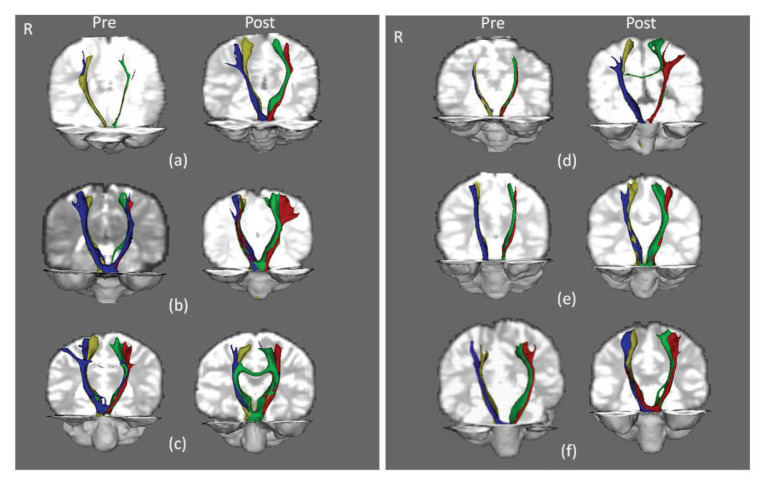
Diffusion images of the CST for upper limb and lower limb overlaid on each subject’s diffusion-weighted brain images. Brain image on the left shows representation of probabilistic water diffusivity in the CST preintervention while images on the right side were acquired after 6 months of exercise therapy intervention. Blue: right CST for left upper limb; Yellow: right CST for left lower limb; Red: left CST for right upper limb; Green: left CST for right lower limb

Post-hoc comparison using Bonferroni test adjusted for multiple comparisons revealed that the post-intervention connectivity index was higher than pre-intervention (*P* = 0.009), while the significant difference in connectivity index among the four tracts were between left lower limb and right upper limb (*P* = 0.03). To determine the CST of which area was significantly increased post-intervention, we performed post-hoc paired *t*-tests corrected for multiple comparisons (significance level *P* < 0.01); this showed that the increase was significant at the right upper limb, *t*(5) = −3.8, *P* = 0.007, *r* = 0.86 (Figure 4). Figure 5 shows the representation of probabilistic water diffusivity in the CST pre- and post-intervention.

## Discussion

The present study utilised a full spectrum of strength training aimed at improving strength of the majority of muscle groups. Our results show that 6 months guided exercise therapy using minimal equipment could increase muscle strength of CP children and improve their movement abilities in all the GMFM dimensions. These findings were consistent with previous studies. Verschuren et al. (17) showed that 6 weeks training of 3–4 sessions per week improved CP children’s aerobic capacity and muscle performance of the lower extremities. Similarly, a 6 weeks randomised controlled trial of exercise intervention showed improvement in gross motor function for non-ambulant children with CP (18). These researchers demonstrated that 6 weeks exercise intervention led to significant short-term improvement in motor function compared to standard physiotherapy care. Six weeks is the minimum time that is generally agreed to have minimal improvement in terms of performance for both normal person and CP patients (19, 20). Nevertheless, to see meaningful or significant improvement in strength among children with CP, sufficient intensity with longer interventions may be needed (21).

The recommended duration of therapy to have better effect remain unknown. Most researchers recommend that exercise therapy is studied for a longer period of time, for example 12 weeks (21, 22). The National Strength and Conditioning Association (NSCA) guidelines recommended 2–3 sessions per week for 8–20 weeks of exercise intervention (19, 20) to allow for adequate recovery between sessions (23). Thus, in the present study the exercise therapy was conducted twice per week for 6 months, under the guidance of a trained instructor.

Previous researchers who aimed to improve motor abilities of CP commonly focus on lower extremities using simple and small number of exercise regime (12, 19), for example, multi-joint exercises such as front and lateral step-ups, half squats and sit-to-stand. Nevertheless, some researchers suggest to start with single-joint resistance exercise such as leg press and heel raises because it is more effective for CP patients compared to multi-joint exercise (21).

Similar to the findings by Englander et al. (8), our study shows that the improvement in motor abilities is accompanied by increased connectivity in the CST, motor pathways from the primary motor area in the precentral gyrus descending down to pons and spinal cord to supply the peripheral limbs. However, the children in Englander et al. (8) study also received autologous cord blood transfusions in conjunction with standard physical and occupational therapies. The connectivity changes are evident of neuplasticity, the ability of neurons to change their function, structure or chemical profile such as quantities and types of neurotransmitters produced (24). There are three main mechanisms of neuroplasticity i.e. habituation, experience-dependent plasticity and cellular recovery after injury (25). Experience-dependent plasticity or sometimes called use-dependent or activity-dependent plasticity involves learning and memory (23). The mechanism process is complex and requires persistent, long-lasting changes in the strength of synapses between neurons and also within neural networks (26).

Previous imaging studies have shown variable abnormal patterns of sensory and motor tracts in CP (27). Damage or injury to white matter is usually related to spastic diplegia and quadriplegia (28). CST and somatosensory radiation tracts are very prone to injury because both tracts mature faster than other white matter tracts (11). Injury to upper motor neurons may decrease cortical input to the reticulospinal and CST that decrease motor unit effectiveness and motor control, causing muscle weakness and abnormal muscle control (29). Abnormalities of muscle tone, spasticity and motor abilities are closely related to the loss of CST.

In our study, 6 months of guided exercise therapy was able to modulate the brain’s connectivity and manifesting as improvement in the execution of movement, as evidenced by the significantly improved GMFM scores. These results were obtained in a sample of unilateral and bilateral CP children with varying age, gender, as well as variable type and extent of brain lesions. Utilising a within-subjects design, each subject was his or her own control and our results showed improvement in GMFM and brain connectivity across all subjects.

Plasticity and brain reorganisation of the young human brain after unilateral injuries have been shown to be occur whereby ipsilateral projections from the non-insulted hemisphere replace the contralateral projections from the lesioned side (30). While this is seen only when the insult to the brain occurs early in development, it opens up the possibility of the the restorative capacity of the brain to reorganise itself. In the adult brain, genetic factor may affect neural plasticity and motor learning progress, for example, polymophisms of the gene for brain-derived neurotrophic factor (BDNF) (31). Synthesis of new proteins, growth of new synapse or modification of existing synapses are prerequisites of activity-dependent plasticity (32). This can be achieved by repetitive brain stimulation such as by transcranial magnetic stimulation (33) or deep brain stimulation (34). Our study shows that exercise therapy is a potential stimulus deemed capable of triggering its reorganisation.

Limitations of this study include small sample size and single arm study design. Further studies with larger population are required to confirm the effects of this relatively affordable and accessible rehabilitation in children with more advanced disabilities.

## Conclusion

This study, although conducted in a limited sample size, gives initial scientific evidence on the benefit of guided exercise therapy to improve motor abilities in CP children concurrent with improvement in white matter connectivity. The exercise therapy is affordable and can be implemented as a home-based training that could bring a lot of benefits not only to strengthen the muscles, but also to maintain it.

## Figures and Tables

**Table 1:** Patients’ demographics

Subject	Age	Sex	Type of CP
1	13	Male	Unilateral
2	18	Male	Unilateral
3	10	Male	Bilateral
4	8	Male	Bilateral
5	10	Female	Bilateral
6	18	Female	Bilateral
7	14	Male	Unilateral
8	14	Female	Bilateral
9	8	Female	Unilateral
10	14	Male	Bilateral

**Table 2:**
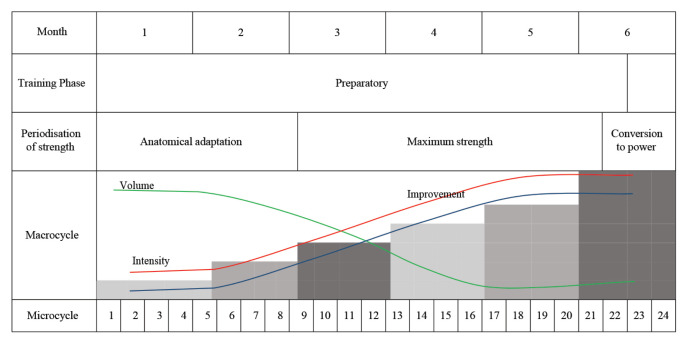
Periodisation table

**Table 3:** Pre- and post-GMFM-88 scores

Dimension	Pre	Post	*P*-value
Lying and rolling	60.2 ± 1.66	65.4 ± 1.97	0.001
Sitting	62.9 ± 1.22	67.8 ± 1.44	0.001
Crawling and kneeling	75.1 ± 1.13	78.2 ± 0.98	0.001
Standing	52.1 ± 1.29	57.2 ± 1.67	0.002
Walking, running and jumping	44.4 ± 1.05	47.6 ± 1.07	0.001

Notes: Post-hoc paired *t*-test, one-tailed, adjusted for multiple comparisons at significance level 0.01. Value is mean ± SEM
